# Beneficial Proapoptotic Effect of Heterobasidion Annosum Extract in Colorectal Cancer Xenograft Mouse Model

**DOI:** 10.3390/molecules28031352

**Published:** 2023-01-31

**Authors:** Anna Sadowska, Diana Sawicka, Katarzyna Godlewska, Katarzyna Guzińska-Ustymowicz, Ewa Zapora, Emilia Sokołowska, Halina Car

**Affiliations:** 1 Department of Experimental Pharmacology, Medical University of Bialystok, Szpitalna 37, 15-295 Bialystok, Poland; 2 Department of Haematology, Medical University of Bialystok, M. Skłodowskiej-Curie 24A, 15-276 Bialystok, Poland; 3 Department of General Pathomorphology, Medical University of Bialystok, Waszyngtona 13, 15-269 Bialystok, Poland; 4 Department of Silviculture and Forest Use, Institute of Forest Sciences, Bialystok University of Technology, Wiejska 45E, 15-351 Bialystok, Poland

**Keywords:** apoptosis, *Heterobasidion annosum*, colon cancer

## Abstract

Fungal extracts possess potential anticancer activity against many malignant neoplastic diseases. In this research, we focused on the evaluation of *Heterobasidion annosum* (HA) extract in colorectal cancer in an in vivo model. The mice with implanted DLD-1 human cancer cells were given HA extract, the referential drug—5-fluorouracil (5FU), or were treated with its combination. Thereafter, tumor volume was measured and apoptotic proteins such as caspase-8, caspase-3, p53, Bcl-2, and survivin were analyzed in mice serum with an ELISA assay. The Ki-67 protein was assessed in tumor cells by immunohistochemical examination. The biggest volumes of tumors were confirmed in the DLD-1 group, while the lowest were observed in the population treated with 5FU and/or HA extract. The assessment of apoptosis showed increased concentrations of caspase 8 and p53 protein after the combined administration of 5FU and HA extract. The levels of survivin and Bcl-2 were decreased in all tested groups compared to the DLD-1 group. Moreover, we observed a positive reaction for Ki-67 protein in all tested groups. Our findings confirm the apoptotic effect of extract given alone or with 5FU. The obtained results are innovative and provide a basis for further research concerning the antitumor activity of the HA extract, especially in the range of its interaction with an anticancer chemotherapeutic agent.

## 1. Introduction

Colorectal cancer is one of the most common malignancies in the human population. Its incidence and mortality rates vary considerably around the world [1]. The greatest chance to achieve remission is provided by radical surgical procedures, chemotherapy, and radiotherapy—depending on tumor localization and its progression. Currently, our chemotherapy schedules are based mainly on 5-fluorouracil (5FU). The drug acts via the suppression of DNA synthesis by restraining the thymidylate synthase. Consequently, the decrease in DNA synthesis through 5FU activates the process of apoptosis. Thus, the effect of 5-FU on cell growth arrest and apoptosis is attributed to the ability of this drug to increase the level and activity of the tumor suppressor p53. 

Oncological treatment complications represent an important problem. The most affecting side effects associated with 5FU treatment comprise mucositis, cardiac toxicity and myelosuppression [2,3,4]. Therefore, there is a challenge for future medicine to search for new drugs which would effectively inhibit the progression of cancer disease but not disrupt the function of normal tissues. The literature data reveal a potential anticancer activity of some fungal extracts. They have enormous therapeutic effects against many cancer diseases acting through different mechanisms. Mushrooms’ medicinal properties may be determined by different types of bioactive compounds and secondary metabolites, such as polysaccharides, lectins or terpenoids. It is claimed that these metabolites confer several pharmacological activities through immune system stimulation and also modulate cellular responses by interfering with particular pathways. For the most part, the suppression of cancer disease is associated with stimulating apoptosis and damaging the DNA of cancer cells [5]. 

The concept of mushroom treatment dates back to a few thousand years ago in traditional Chinese medicine. The beneficial properties of some herbal and fungal species were also valued in other countries of Asia later on [6,7,8]. Medical treatment based on fungi or compounds derived from fungi has been raised to the scientific rank and named mycotherapy or fungotherapy [9]. A large number of mushroom species have never been analyzed regarding their antitumor activity. The features of medicinal mushrooms indicate that even being producers of several substances, they can be involved in various cancer-related processes synergistically when used as a treatment. Therefore, experiments on anticancer effects triggered by combinations of molecules in their extracts, and not only particular fungi derivative compounds, receive a great deal of scientific attention [8]. Ongoing studies report on the possible anticancer activity of extracts obtained from arboreal fungi. They have great therapeutic potential against many types of cancer, acting through various mechanisms. Most often, inhibition of the tumor process is associated with the induction of apoptosis and DNA damage in cancer cells [10,11]. The prevalent mechanisms of their activity are the inhibition of cancer proliferation, the induction of cancer phagocytosis, and cell cycle arrest. Apoptosis induction is mainly mediated through the regulation of expression of p53, p27, caspases 3, 8, 9, and the Bax/Bcl-2 ratio [10]. Crucial substances derived from medicinal mushrooms include schizophylan, lentinan, krestin polysaccharide (PSK), and polysaccharidopeptide (PSP). Their main mechanism of action is associated with the enhancement of cell-type immune response, including the activation of macrophages, NK cells, and cytotoxic T lymphocytes. They have no cytotoxic properties in normal cells, including monocytes and macrophages [12]. *Heterobasidion annosum sensu lato* (s.l.) (HA) is a basidiomycete fungus considered to be a highly destructive conifer pathogen causing root rot in forests in the northern boreal and temperate hemisphere. The main host of HA in northern Europe is Scots pine (*Pinus sylvestris*). It also infects other conifers, such as junipers and spruces, and various broad-leaved trees. HA typically kills pine tree roots, causing sudden tree mortality due to the acquisition of nutrients [13,14,15,16,17,18]. While other authors describe the phylogeny and pathogenicity of HA, we try to find other possibilities concerning the biological properties of this fungus.

In our previous study, we examined the possibility of HA extract application in medical use by testing its cytotoxic activity against the DLD-1 colon cancer cell line [19]. Hence, in this paper, we expanded our research to investigate the influence of this fungal extract on cancer tumors in mice with implanted xenografts and its comparison to the anticancer drug 5FU. We further evaluated the assessment of the apoptotic process in the sera of mice treated with HA extracts applied alone or in combination with 5FU. Therefore, our study aims to determine a possible interaction of HA with the referential drug to assess the potential to support or disqualify its combined application.

## 2. Results and Discussion

### 2.1. Evaluation of Tumor Volume

**Figure 1:**
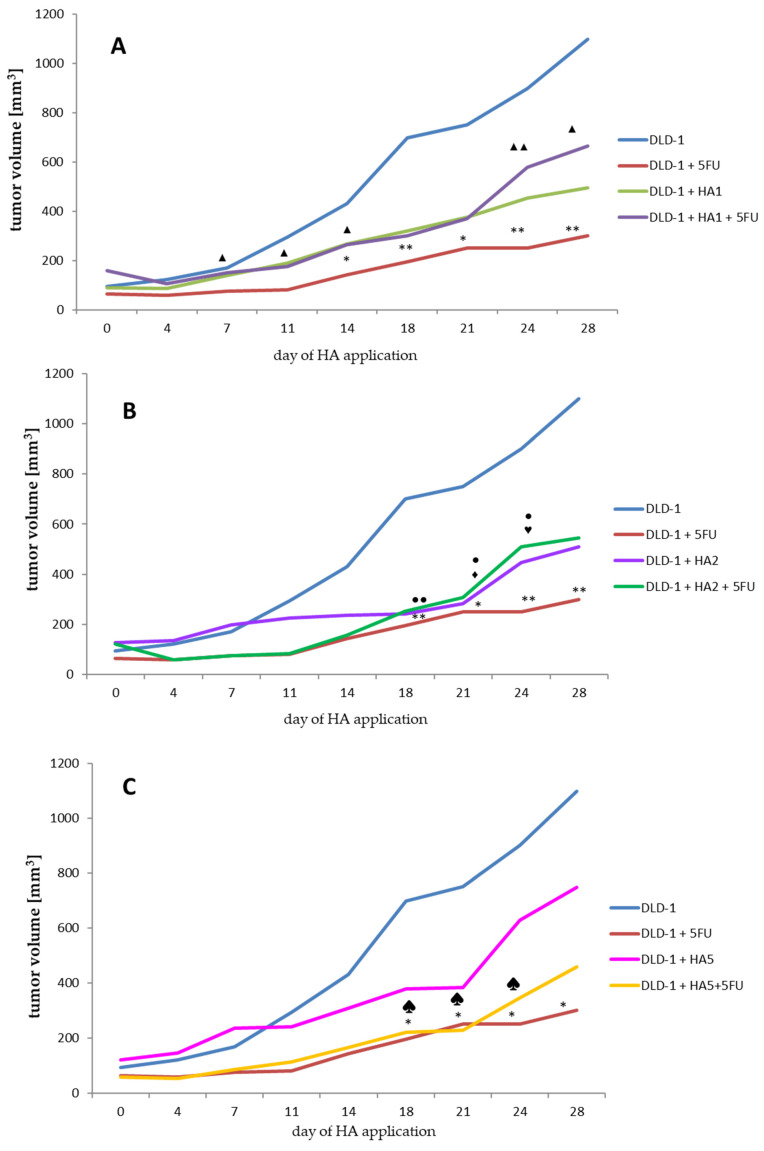
Analysis of tumor volume changes (**A**–**C**) in study groups. Significance in tumor volume is referred to the same day of the experiment between groups (**A**–**C**). (**A**–**C**). * *p* < 0.05 DLD-1 vs. DLD-1 + 5FU, ** *p* < 0.01 DLD-1 vs. DLD-1 + 5FU. (**A**) ▲ *p* < 0.05 DLD-1 + 5FU vs. DLD-1 + HA1 + 5FU, ▲▲ *p* < 0.01 DLD-1 + 5FU vs. DLD-1 + HA1 + 5FU. (**B**) ● *p* < 0.05 DLD-1 vs. DLD-1 + HA2, ●● *p* < 0.01 DLD-1 vs. DLD-1 + HA2—♦ *p* < 0.05 DLD-1 vs. DLD-1 + HA2 + 5FU, ♥ *p* < 0.05 DLD-1 + 5FU vs. DLD-1 + HA2 + 5FU. (**C**) *♠ p* < 0.05 DLD-1 vs. DLD-1 + HA5 + 5FU.

The first step of our study was to assess the changes in tumor size after each measurement in all eight groups of mice with inoculated DLD-1 cells (Figure 1 and Figure S1, Table S1). On the last day of the experiment, the largest tumor volume was found in a group of mice with implanted DLD-1 colon cancer cells. After exposure to the 5FU reference drug, a significant reduction in tumor growth was noted. This result also proves that the dose and frequency of 5FU administration were correctly selected.

More particularly, tumor volume measured in the DLD-1 + 5FU group was about 3.7 times smaller compared to the DLD-1 group at the end of the experiment. 

Based on our previous experiments, where we analyzed acute oral toxicity and performed a preliminary investigation on a xenograft tumor model with only one extract dose, we decided to extend the concentration range to 1, 2 and 5 mg/kg bw. 

In a group where the lowest dose of HA1 (1 mg/kg bw) (Figure 1A) was used, the tumor volume at the end of the experiment was more than 2-fold smaller compared to the DLD-1 group. It is interesting that in mice receiving 5FU and HA1 extract, no significant reduction in tumor volume was observed (the tumor volume was about 1.5 times smaller compared to the DLD-1 group), suggesting that this dose of extract does not interact with 5FU to increase the anti-tumor effect. Doubling the dose of HA extract (HA2, 2 mg/kg bw) (Figure 1B) inhibited tumor growth from day 7, but showed significant suppression from day 18 in comparison to the DLD-1 group. After the combined administration of HA2 extract and 5FU, the mean size of the tumor was similar to that observed after the administration of HA2 extract alone. This result indicates the high potential of HA extract regarding antitumor activity in colorectal cancer. The application of HA extract at a dose of 5 mg/kg bw (HA5) caused a nonsignificant growth rate decrease in tumor size, while the combined administration of this dose with 5FU significantly reduced the tumor size from the day of the first application to day 21, and a significant reduction of tumor volume growth was obtained from day 18 to 24 of HA5 + 5FU (Figure 1C). This indicates a favorable interaction of the above dose with 5FU. The tumor growth rate estimated on the basis of its volume points to a similar tendency in all concentrations of the extract. Mean volumes were calculated at the end of the experiment, and for the DLD-1 + HA1, DLD-1 + HA2 and DLD-1 + HA5 groups amounted to 494.77, 510.25 and 749.22 mm^3^, respectively. The volumes were bigger when compared to the DLD-1 + 5FU group (300.45 mm^3^), but smaller than the combined effect of 5FU and HA extract in groups DLD-1 + HA1 + 5FU and DLD-1+ HA2 + 5FU (664.91, 545.13, respectively) (Table S1). It is noteworthy that volumes of tumors started to grow more rapidly since day 21 of the experiment, which might suggest a need to shorten the HA application to avoid further tumor growth. Furthermore, we noticed the shrinkage of tumors until they disappeared completely at the end of the experiment and that affected the animals (up to 30%) from the HA1 and HA2 groups. This prompts us to assess and compare the parameters presented here after 21 days of an experiment to confirm or exclude resistance and/or adaptation to HA extract. 

Summing up this part of the research, it can be stated that the anti-tumor effect of HA extract might be dependent on the dose used, however, more diversified doses would help determine its potential more accurately. An important result of the study is the absence of adverse interactions with 5FU, which confirms the lack of significance between the size of the colon tumor after administration of 5FU only and 5FU with the HA extract.

### 2.2. Histological and Immunohistochemical Examination

Tumor necrosis is a characteristic property of solid tumors as an effect of chronic ischemic injury owing to the rapid growth of tumors. The size of necrosis is considered to express the extent of hypoxia in tumors [20,21]. Moreover, increased hypoxia is associated with metastatic potential, worse prognosis in tumors and greater resistance to chemo- and radiotherapy [20,22,23].

Since tumor necrosis is an indicator of particularly aggressive tumors, in recent publications, necrosis has been identified as an important prognostic marker of survival in a variety of tumors, such as bowel [23], kidney [24], lung [20], pancreatic [25], and breast cancer [26].

From a histological point of view, necrotic tissue differentiates from viable cells by its morphology. It has been described that necrotic cells resemble “moth-eaten” as they undergo a continuous disintegration. The nuclei in necrotic tissue are often shrunk or are completely absent, whereas viable tissue usually shows well-pronounced nuclei. Moreover, necrosis is often associated with an inflammatory response to remove dead cells [27]. 

Apoptosis and necrosis have usually been reported as two separate pathways of cell death. However, a great number of studies have described the conversion of apoptosis to necrosis, which means that apoptosis can be exchanged with necrosis because of acute disruptive factors, and the ATP concentration is also of crucial significance in the process. Higher concentrations of ATP are favorable to the apoptotic program, whereas more than 70% of ATP loss triggers necrosis. Owing to this fact, the reduction of ATP reaching a specific level causes either the shift of cell death from apoptosis to necrosis or induces direct necrosis. Moreover, it was noticed that the attributes characteristic of apoptosis are accompanied by those typical of necrosis, which signifies that the transition from apoptosis to necrosis might occur after the apoptotic process begins. Some factors favoring the switch have been revealed, such as H_2_O_2_, one of the most common ROS (reactive oxygen species), which activates apoptosis in average concentrations while causing necrosis at higher levels. The conversion from apoptosis to necrosis was also associated with the c-myc or bcl-2 expression under hypoxia, which was related to the ATP levels [28,29,30]. 

**Figure 2:**
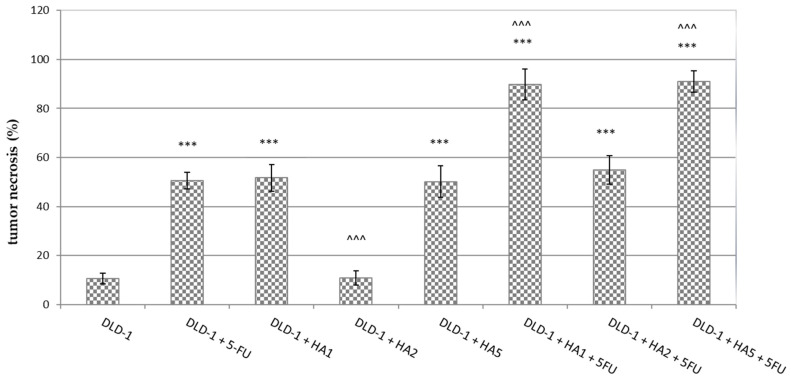
Tumor necrosis in CRC tumors from experimental groups. The results are presented as the mean ± SD for each group. *** *p* < 0.001 vs. DLD-1; ^^^ *p* < 0.001 vs. DLD-1 + 5FU.

The analysis of histopathological slides showed the highest levels (about 90%) of necrosis in DLD-1 + HA1 + 5FU and DLD-1 + HA5 + 5FU groups. We observed significant differences in tumor necrosis between the tested groups vs. DLD-1, except for DLD-1 + HA2, where the results were comparable to those observed in the DLD-1 group (Figure 2). Statistical significance was also observed in DLD-1 + HA2, DLD-1 + HA1 + 5FU and DLD-1 + HA5 + 5FU groups when we compared the degree of tumor necrosis to DLD-1 + 5FU. It is interesting that the percentage of tumor necrosis induced by 5FU is comparable to that induced by HA1 and HA5. Additional experiments might be helpful to explain these results in terms of whether the extract could reduce tumor growth alone better than the combined administration of these two compounds, or if its effect is similar to 5FU. It cannot be ruled out that in the groups with a high percentage of necrosis at the end of the experiment, the process of apoptosis changed into necrosis, especially after 21 days. when we noticed a significant growth of tumors. That could have contributed to the development of hypoxia, which in turn may reflect the percentage of necrosis in tumors as well as reduce the response to chemotherapy [20,21].

**Figure 3:**
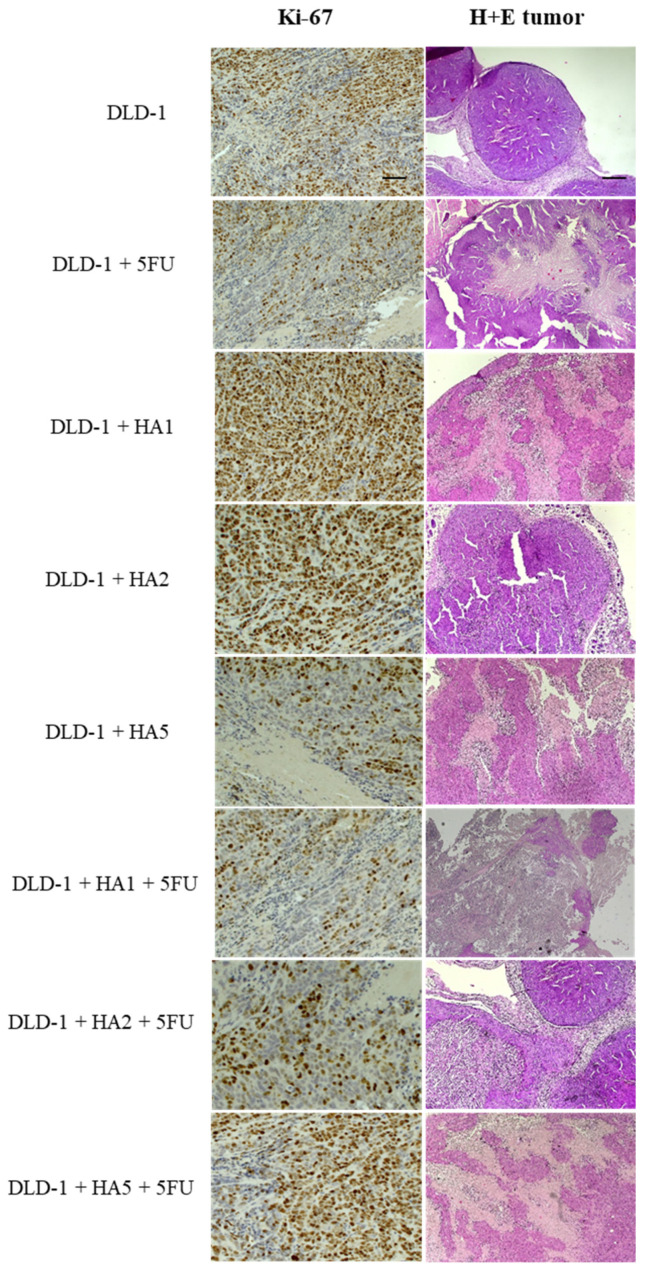
H + E staining and Ki-67 immunochemical expression in DLD-1 tumors of mice (*n* = 6 per group); (Ki-67—100× magnification, scale bar = 50 µm; H + E staining—40× magnification, scale bar = 20 µm).

**Figure 4:**
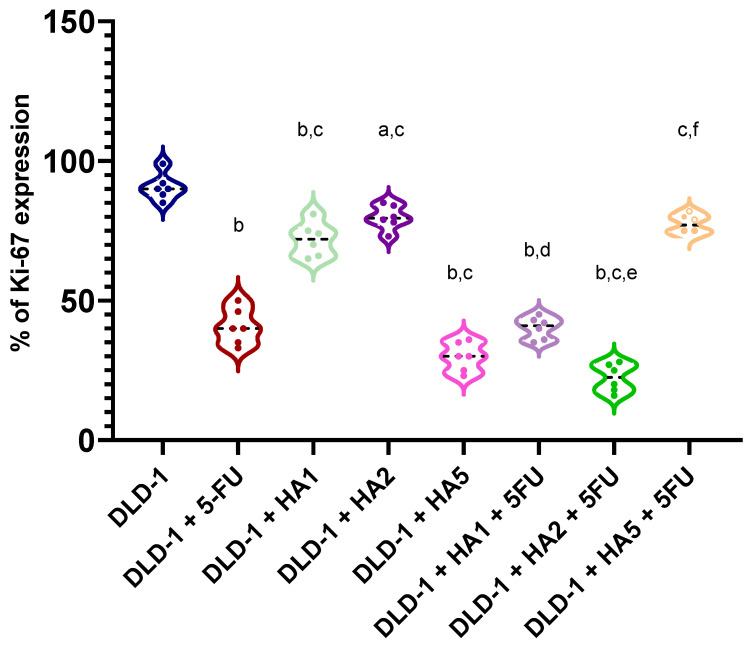
Expression of Ki-67 in tumors of mice (*n* = 6 per group). ^a^ *p* < 0.05 vs. DLD-1, ^b^ *p* < 0.001 vs. DLD-1, ^c^ *p* < 0.001 vs. DLD-1 + 5FU, ^d^ *p* < 0.001 vs. DLD-1 + HA1, ^e^ *p* < 0.001 vs. DLD-1 + HA2, ^f^ *p* < 0.001 vs. DLD-1 + HA5.

In tumors of mice, we also assessed the expression of the Ki-67 protein (Figure 3 and Figure 4). Ki-67 is an antigen expressed in the nucleus of proliferating cells. Some studies point to its predictive role in human malignancies, such as gastrointestinal, breast and prostate tumors [31,32,33,34,35,36]. The Ki-67 antigen occurs in the cell nucleus, while the Ki-67 protein can be detected in all active phases of the cell cycle and is absent in resting cells (G0), which makes it a notable marker of growing tumor cells [37]. It was also reported that Ki-67 expression was significantly higher in tumor tissues compared to peritumoral tissues, and its levels were notably associated with lymph node metastasis and the poor prognosis of colorectal cancer [38]. However, the results concerning the prognostic role of Ki-67 in CRC were partly contradictory [36,39,40]. Our results determined in tumors showed a positive reaction of Ki-67 in all tested groups. Nonetheless, the highest percentage of Ki-67 expression was noted in the DLD-1 group, and the results showed statistically significant differences compared to other groups, except for DLD-1 + HA5 + 5FU. It is important to clarify that the DLD-1 cell line is derived from a patient with colorectal adenocarcinoma in Dukes’ stage C with regional lymph node metastasis and is characterized by a high degree of proliferation. As indicated in Figure 1, the 21st day of the experiment was critical, and in all experimental groups we observed a sharp increase in tumor volume, which might be the explanation for a positive expression of Ki-67 in tumors excised at the end of the experiment.

Li K et al. [41] performed a study investigating the anticancer effect of ethanolic extracts of *Ganoderma lucidum* (BSGLEE) sporoderm-broken spores. The oral gavage of BSGLEE in concentrations of 75 and 150 mg/kg inhibited HCT-116 xenograft tumor growth in nude mice, which was also shown by suppressed Ki-67 staining. The authors concluded that a dramatic decrease in Ki-67 staining suggests a reduction of tumor proliferation after BSGLEE treatment; however, constant tumor growth during the experiment was still notable [41]. Similarly, Jedinak et al. [42] showed that *Pleurotus ostreatus* in a dose of 500 mg/kg significantly reduced Ki-67 expression, though cancer in mice was induced by the administration of 2-amino-1-methyl-6-phenylimidazo [4,5-b] pyridine, a food-borne carcinogen [42]. Studies performed by Sawicka et al. [43] showed the expression of Ki-67 in tumors of mice receiving imidazolium salts substituted with lithocholic acid (S6) and/or 5FU. The results presented the expression of about 80% in the DLD-1 group, while all groups treated with chemotherapeutic agents (S6 or 5-FU) were similar and was estimated to be about 60% [43].

In summary, our results showed that substances administered to mice induced tumor necrosis, both in the tested extract and reference drug, with an even better combined effect of 5FU and HA. However, it has been established that tumor necrosis may be the result of two distinct pathways, one of which involves apoptosis, while the other has its background in the stimulation of the inflammatory pathway through rapid tumor growth, leading to vascular insufficiency and tissue hypoxia [44].

### 2.3. Parameters of Apoptosis

In order to evaluate the mechanism of the tested HA extract, more detailed parameters were surveyed to allow the assessment of its antitumor activity in the field of apoptosis.

Apoptosis is a form of programmed cell death that plays a fundamental role in a variety of biological processes, including the elimination of unwanted, harmful or mutated cells [45,46]. Characteristic features of the apoptotic process, i.e., cell shrinkage, membrane blebbing, nuclear and cytoplasmatic condensation or fragmentation of DNA are mostly triggered in the execution phase by caspases. Caspases are a family of cysteine proteases, synthesized as inactive zymogens and activated through proteolytic cleavage in both mitochondrial and death receptor–mediated apoptotic pathways. Caspases are central to the mechanism of apoptosis, as their family consists of two groups, the initiator and effector caspases. Following their activation, the initiator caspases go along to activate the executioners [45,47,48].

Two main apoptotic pathways can be distinguished: the extrinsic pathway and the intrinsic or mitochondrial pathway. However, there is currently evidence that both paths are connected and that molecules in one path can affect the other [49]. External signaling pathways that initiate apoptosis include the transmembrane receptor–mediated interactions. These involve receptors that are members of the tumor necrosis factor (TNF) superfamily [50] and lead to the cleavage and activation of initiator caspases 8 and 10, which in turn cleave and activate executioner caspases 3, 6, and 7, causing apoptosis [51].

The intrinsic signaling pathways that initiate apoptosis include a diverse range of non-receptor stimuli producing intracellular signals acting directly on cells. These pathways are initiated in cellular mitochondria when cytochrome c is released. Factors stimulating the intrinsic apoptotic pathway include radiation, toxins, hypoxia, hyperthermia, viral infections and free radicals. All these stimuli initiate changes in the inner mitochondrial membrane, which cause mitochondrial permeability transition (MPT), loss of mitochondrial transmembrane potential, and the release of proapoptotic proteins from the inter-membrane space to the cytosol [52]. Subsequently, Apaf-1 formation of the apoptosome leads to caspase 9 activation followed by the activation of the executioner caspases 3, 6, and 7 [51]. The process of apoptosis can be inhibited by a specific group of proteins called apoptosis inhibitors. A key member belonging to the family of inhibitors of apoptosis (IAP) is survivin. Its overexpression suppresses the extrinsic and intrinsic apoptosis pathways, thus apoptosis is stimulated when survivin is depleted [53].

**Figure 5:**
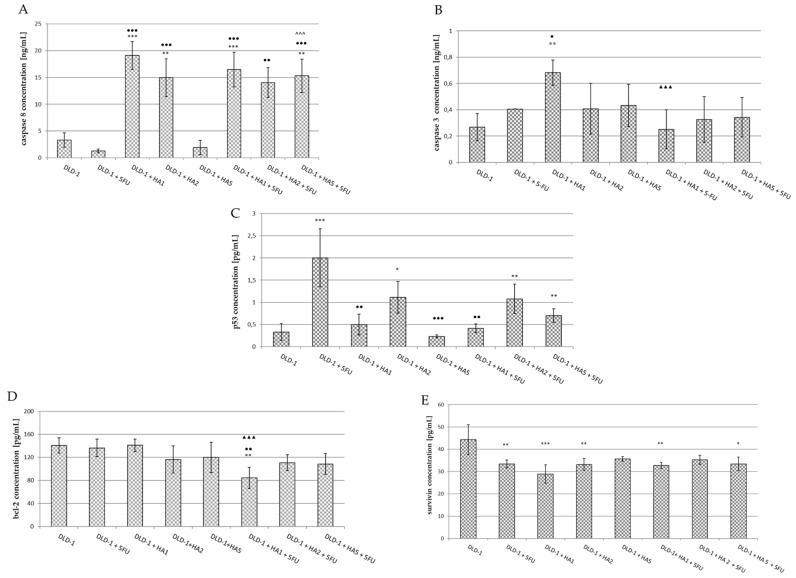
Caspase 8 (**A**), caspase 3 (**B**), p53 (**C**), Bcl-2 (**D**) and survivin (**E**) concentrations in serum of mice from experimental groups. The results are expressed as the mean ± SD for each group. * *p* < 0.05 vs. DLD-1; ** *p* < 0.01 vs. DLD-1; *** *p* < 0.001 vs. DLD-1; ● *p* < 0.05 vs. DLD-1 + 5FU; ●● *p* < 0.01 vs. DLD-1 + 5FU; ●●● *p* < 0.001 vs. DLD-1 + 5FU; ^^^ *p* < 0.001 vs. DLD-1 + HA5; ▲▲▲ *p* < 0.001 vs. DLD-1 + HA1.

In our study, we focused on the analysis of selected apoptotic parameters, i.e., caspase-8 and -3, the p53 protein, Bcl-2, and survivin, which were assessed in the serum of mice from experimental groups (Figure 5).

The recruitment and cleavage of pro-caspase 8 to generate the active form of caspase 8 is a crucial biochemical event in death receptor-mediated apoptosis, as caspase 8 is considered to be the main mediator of the extrinsic pathway [54]. There are about three different mechanisms engaged in caspase activation; however, the best understood occurs through the recruitment of complexes adjacent to a membrane which is associated with death receptors [45]. In our study, the assessment of caspase 8 showed the lowest concentration in mice after the i.p. administration of 5FU (1.286 ± 0.32 ng/mL); however, the differences were not statistically significant (Figure 5A). In the group of animals receiving HA extract at the lowest dose (DLD-1 + HA1), a significant increase in caspase 8 concentration was found compared to the results in the DLD-1 group and the group receiving only 5FU. This also corresponds to smaller tumor size in this group of animals. In addition, it has been observed that 5FU does not change the effect of HA1 extract when used in conjunction; in other words, the observed effect on caspase 8 is related to the HA extract only. The occurring interaction of the drug and HA allows us to conclude that the HA extract has a paramount effect over 5FU, but it is also likely that HA changes the effect of 5FU in the range of caspase 8 activation. The intensification of caspase 8 activity was also noted after the administration of HA extract at a dose of 2 mg/kg bw (vs. DLD-1 and DLD-1 + 5FU groups). In contrast, in the serum of mice receiving HA2 extract together with 5FU (DLD-1 + HA2 + 5FU), a significant increase of caspase 8 concentration was noticed compared to the 5FU group, but there was no difference in relation to the group receiving only the HA extract at the same dose (DLD-1 + HA2). These results suggest a change in the effect of 5FU and the activation of caspase 8 dependent apoptosis under the influence of the HA extract. In mice receiving HA extract at a dose of 5 mg/kg bw a serum caspase 8 concentration was comparable to that of the DLD-1 group, whereas it was significantly higher when the extract in the above-mentioned dose was used together with 5FU (vs. DLD-1, DLD-1 + 5FU and DLD-1 + HA5 + 5FU). The obtained results imply a positive interaction of HA extract with 5FU with a mechanism potentially related to caspase 8 activation. However, an explanation of the specific mechanisms by which caspase 8 increased concentrations after the administration of the HA extract and also its interaction with 5FU is not possible on the basis of the current research and requires further experiments.

The executive caspase 3 activates both pathways of the programmed cell death process triggering the same effect, i.e., the proteolytic cleavage of procaspase 3. The conversion of procaspase 3 to active caspase 3 results in a cascade production of active executive enzymes that catalyze the hydrolysis of many proteins [55,56]. Caspase 3 concentration measured in the serum of mice was the highest in the DLD-1 + HA1 group, and only these results were statistically significant compared to DLD-1, DLD-1 + 5FU and DLD-1 + HA1 + 5FU groups (Figure 5B). Flanagan et al. [57] examined caspase 3 activity in the serum of patients with metastatic colorectal cancer. They showed the highest levels in patients with progressive disease and the poorest outlook, while those patients who responded to therapy had significantly lower levels of serum caspase 3. That indicated the influence of active caspase 3 on disease progression. The results indicated caspase 3 to be a potential predictive marker of response to 5FU-based chemotherapy in advancer colorectal cancer [57].

Summing up, the effect of HA extract at a dose of 1 mg/kg bw may be dependent on caspase 8 and 3 activation. Based on this data, we suggest that the extrinsic pathway of apoptosis is activated, but the detailed mechanism requires further study. Importantly, in the DLD-1 + 5-FU group of mice, we observed the smallest average tumor size, which allows us to assume that this drug belonging to the group of antimetabolites, and interfering with the synthesis and stability of DNA and RNA nucleic acids, does not affect the process of apoptosis through caspase 8; however, we cannot exclude the impact on the executive caspase 3, because we observed a small increase in its concentration. This thesis is confirmed by the results of Mhaidat et al. [58]. The authors conducted experiments to evaluate the in vitro cytotoxic effect of 5-FU against a panel of CRC cells. The analysis of caspase cascade activation revealed that the activation of caspase 9 is the initiating event, and that establishes caspase 9 as the apical caspase in the 5FU-induced apoptosis. Caspase 9’s similarly to caspase 8 is generally considered to be the initiator factor in chemotherapy-induced apoptosis [58]. Yang L. et al. [59] studied the activity of 5FU in hepatocellular carcinoma (HCC) in the field of apoptosis. Due to the high resistance of tumor cells to standard chemotherapy regimens, researchers combined 5FU and andrographolide (ANDRO), a bicyclic diterpenoid lactone isolated from Andrographis paniculata. The synergistic induction of apoptosis was observed due to 5-FU + ANDRO polytherapy, which was confirmed by the increased activity of caspase 8, p53 and significant changes in Bax conformation in cancer cells.

The p53 protein is one of the best known tumor suppressor proteins encoded by the TP53 suppressor gene located on the short arm of chromosome 17 [47]. It can be activated in response to DNA damage, oncogene activation, or hypoxia [60]. P53 is called the “genome protector”, as it is responsible for the induction of apoptosis, cell cycle regulation, gene amplification, DNA recombination, chromosome segregation, and cellular senescence [61]. Defects in the p53 tumor suppressor gene can be associated with more than 50% of human cancer cases. It has been demonstrated that the loss of the p53 function reduces cellular sensitivity to 5-FU [62]. However, depending on the cell damage degree, p53 may contribute to apoptosis or DNA defect correction by cell cycle arrest [23]. It has been said that p53 serves as the major route for the anticancer effect of 5FU and determines the cellular sensitivity to cytotoxic 5FU impact [63,64], and in our study, we also obtained a significant increase in the serum of mice receiving 5FU (Figure 5C). This result indicates the proper dose and dosage of 5FU used in our experiment. In this group (DLD-1 + 5FU) tumor size was the smallest on the last day of the experiment compared to other studied groups. The above results may suggest that 5FU, by damaging nucleic acids in cells, activates the p53 protein leading to apoptotic cell death. However, more precise measurement in tumor tissue is required to confirm this speculation. In the serum of mice receiving the HA extract at doses of 1 and 5 mg/kg bw alone or together with 5FU, no significant changes in p53 protein concentration were observed. A marked decrease in its concentration was noticed when results in groups DLD-1 + HA1, DLD-1 + HA5, and DLD-1 + HA1 + 5FU were compared to the 5FU group. In the serum of mice receiving HA extract at a dose of 2 mg/kg bw alone or in combination with 5FU, a significant increase in p53 protein concentration was observed only compared to DLD-1, which indicates that HA extract in a dose of 2 mg/kg bw may stimulate apoptosis expressed by a high level of p53 in the serum of mice. To sum up, the decrease in the level of p53 protein after combined administration of HA with 5FU suggests an interaction resulting in diminishing the influence of 5FU on p53 protein. However, the observed decrease in tumor size after the combined application of HA with the drug may result in changing 5FU activity in the above mechanism of apoptosis. Recent studies demonstrated that human ribosomal protein L3 (rpL3) can activate the transcription of p21 in a p53-independent manner. RpL3 acted as a stress-sensing molecule crucial for cell response to 5-FU treatment in colon cancer cells where active p53 is lacking. The results also indicated that rpL3 overexpression could improve the cytotoxic effects of 5-FU, which acts as a DNA damage agent inducing apoptosis. On the contrary, the loss of rpL3 makes the chemotherapeutic effects of this drug ineffective [65,66]. Further studies are necessary to investigate the potential mechanism relative to the rpL3 protein. Balogh et al. [67] reported that mutant p53 in serum is a promising novel parameter for the evaluation of cellular biology and the prognosis of breast cancer using blood samples, thus avoiding surgery. Moreover, it has been stated that the serum assay for p53 oncoproteins using ELISA can be performed easily and repeatedly due to its minimal invasiveness compared with assays using tissue materials [68,69]. It is consistent with our experiments, and thus we aimed to assess if the serum level of apoptotic proteins could be a promising factor evaluating tumor proliferation.

In the present study, the levels of apoptotic inhibitors were also assessed. The main regulators of caspase functions are proteins from the Bcl-2 family. The Bcl-2 protein promoting cell survival belongs to the group of proto-oncogenes, and its action may save the cell from programmed death. Bcl-2 is located in the mitochondria, in the endoplasmic reticulum, and also in the nuclear membrane. The main function of this protein is a balance control between proteins from this group [70].

In our research, significant differences concerning Bcl-2 protein concentration were noted in the DLD-1 + HA1 + 5FU group compared to the DLD-1, DLD-1 + HA1 and DLD-1 + 5FU groups (Figure 5D). The highest concentration was obtained in the DLD-1 group, while in other groups the results were lower; however, no statistical significance was noted. Wang et al. [71] performed a study with an etanolic extract of Pholiota adiposa on hepatocellular tumor-bearing mice. The authors achieved the decreased expression of Bcl-2 protein in IHC as well as western blot analysis [71]. It was also shown that decreased Bcl-2 expression plays an important role in the induction of p53-dependent apoptosis [72]. It has been stated that serum levels of Bcl-2 protein may reflect the degree of its expression in cancer tissue, and while it is easily determinable it could be useful as a prognostic marker in colorectal cancer [73].

Survivin belongs to a family of inhibitors of apoptosis (IAP). It can be found primarily in the cytosol of cancer cells. Cytosolic survivin is thought to act as an apoptotic suppressor, while nuclear survivin regulates cell division. However, the pathological significance of nuclear survivin as a beneficial prognostic marker for cancer cells is still disputable. There is ambiguous data from patient studies indicating the expression of nuclear/cytosolic survivin as an unfavorable or beneficial prognostic marker in cancer [74]. In addition, survivin has also been detected in mitochondria and was shown to be released into the cytosol in response to cellular stress stimuli, suppressing caspase activation [74]. Khan et al. [75] showed that the extracellular form of survivin favors surrounding tumor cells to increase resistance to therapy, multiply rapidly and demonstrate an increased potential for invasion [75]. Although the mechanism of apoptosis inhibition by survivin is still unknown, both direct and indirect binding of survivin to caspases is expected to contribute to its inhibition [76]. It is postulated that the direct binding of survivin to effector caspase 3 plays a key role in this mechanism. Some studies suggest that survivin binds directly to caspase 9 and inhibits its activity [77]. The protein also has the ability to promote angiogenesis in cancer cells by increasing VEGF expression and promoting endothelial cell proliferation through mechanisms that are still unclear [78]. The authors suggest that the serum level of survivin measurable before surgical intervention may be useful as a biomarker [79,80,81]. Our research showed a decrease in the survivin concentration in the serum of mice in all studied groups compared to the DLD-1 group (Figure 5E). It is worthy of note that the decrease in survivin concentration noted after 5FU administration was similar to that obtained in other groups after combined administration of 5-FU with HA extract. These results indicate a significant effect of 5FU and HA extract on survivin concentrations. Yang L. et al. [82] reported the significant effect of 5FU on the induction of apoptosis by survivin-dependent signaling pathways. They found that 5FU increased the sensitivity of hepatocellular carcinoma cells by TRAIL (TNF-related apoptosis-inducing ligand). The results indicate that the induction of apoptosis was stimulated by the increased expression of the DR5 receptor and the reduced expression of survivin [82]. Similarly, our findings suggest that diminished survivin concentration implies the willingness of an organism to respond to the suppression of the apoptotic process. Moreover, when we compare the results from survivin and caspase 3, it gives a consistent view of the aforementioned mechanism pointing to a connection of caspase 3 by survivin.

Several studies performed with other fungal extracts are available in the scientific literature and can be discussed due to the lack of data on the effect of HA. Zhang et al. stated a similar effect of the polysaccharide from the fungus *Grifola frondosa* on the proliferation of breast cancer cells [83]. In that study, the cytotoxic effect of polysaccharides on breast cancer cell lines was demonstrated, as evidenced by a decrease in cell viability and an increase in LDH release, the accumulation of reactive oxygen species, the activation of caspase 3, caspase 8, and the mitochondrial apoptotic pathways. Decreased Bcl-2 and Bcl-xL levels and increased Bax expression were also observed [83]. Proteins from the Bcl-2 family, located in the outer mitochondrial membrane, serve as an important indicator in the mitochondrial apoptotic pathway [84]. In addition, the accumulation of intracellular reactive oxygen species was observed in this study. Their overproduction causes oxidative stress, which also stimulates mitochondrial apoptosis [83]. Lin et al. [85] also demonstrated the proapoptotic effect of polysaccharides obtained from *G. frondosa* on hepatocellular carcinoma cell proliferation [85]. Similarly to our study, Wang et al. [86] showed a reduced level of Bcl-2 and the enhanced activation of caspase 3, caspase 8, and caspase 9 in cordycepin-treated tumor tissues. The effects of cordycepin, a major compound separated from *Cordyceps sinensis*, were evaluated on breast cancer cells in both in vitro and in vivo experiments [86].

A summary of the available knowledge about medicinal mushroom extracts was presented in a study by Tsai et al. [87]. In this publication, the effect of Inonotus obliquus extract on the proliferation of the HCT-116 colon cancer cell line was examined. Cell survival was measured by MTT tests and cell membrane integrity was determined by LDH release. The expression of mRNA was examined by real-time PCR, while p53 and NF-κB levels were assessed using western blotting. In addition, the effect of I. obliquus extracts on HCT-116 cells was assessed based on caspase 3 activity. In analyzing the results, an increased expression of proapoptotic genes (Bax, Bad and caspase 3) and an increase in the Bax/Bcl-2 ratio were found. Moreover, I. obliquus extract induced G0/G1 phase cell cycle arrest. The increased expression of proapoptotic genes (p53, p21) and the decreased expression of genes encoding antiapoptotic proteins (cyclin D1) were found. The extract from *I. obliquus* mycelium significantly increased the expression of p53 protein in HCT-116 cells and stimulated a decrease in the expression of the NF-κB protein and the COX-2 gene in colorectal cancer cells [87].

## 3. Materials and Methods

*Heterobasidion annosum* was collected in September 2013 in the Bialowieza Forest (Hajnowka Forest District) from the trunks of the infected Pinus sylvestris L. Microscopic examination and taxonomic identification of HA fruiting bodies were made by mycologist Marek Wolkowycki. For this purpose, OPTA TECH a binocular magnifier at 10–25× magnification was used. Tubular and context microscopic preparations were also made. An OPTA TECH LAB-40 light microscope with variable phase contrast was used in order to observe the microscopic features. Preparations from fresh specimens were made in water, while from dry samples they were made with 3–5% KOH.

A herbarium specimen was deposited in the Fungarium of the Institute of Forest Sciences of the Bialystok University of Technology.

The chemical composition was confirmed by FTIR, GC-MS and HPLC techniques and was published in our previous study (Supplementary Data) [19].

### 3.1. Preparation of HA Extract

The methanolic extract was prepared as described previously [19]. Briefly, fresh fruiting bodies of HA were cut. The raw material was transferred to bottles with methanol. The samples were stirred, and after 24 h the solvent was decanted and a fresh portion of methanol was added. The extraction was repeated three times. Extracts were then combined and filtered through the paper filter. Methanol was evaporated and dry residues were used for research. For in vivo experiments, HA extract was dissolved in pure DMSO first and diluted with sterile saline (0.9% NaCl) with the final DMSO (Chempur, Piekary Śląskie, Poland) concentration at 2%.

### 3.2. Tissue Culture

The DLD-1 colon cancer cell line derived from human was purchased from American Type Culture Collection. The cells were maintained in RPMI 1640 medium (ATCC) supplemented with 10% fetal bovine serum (FBS) (Eurx, Gdansk, Poland), 50 U/mL penicillin, 50 mg/mL streptomycin (Gibco, Thermo Fisher Scientific, Inc., Waltham, MA, USA) at 37 °C in a 5 % CO_2_ incubator. Cells reached confluence on day 4 and were used for the implantation. The cells were used in the 8th to 14th passages.

DLD-1 is a unique cell line, which possesses high tumorigenic properties. It is characterized by the positive expression of different genes, such as myc+; myb+; ras+; fos+; sis+; p53+, and miR-21, which determine their resistance to treatment by classical chemotherapy [88]. According to ATCC data, it was indicated that this cell line is appropriate for in vivo evaluation in a xenograft model in nude mice.

### 3.3. In Vivo Study

All methods were performed in accordance with the relevant guidelines and regulations. All experimental protocols were approved by the Local Ethical Committee for Experiments on Animals in Bialystok of the Medical University of Bialystok, Poland (no 139/2015) and the Local Ethical Committee for Animal Experiments in Olsztyn, Poland (no 34/2018).

#### Xenograft Studies in Nude Mice

**Figure 6:**
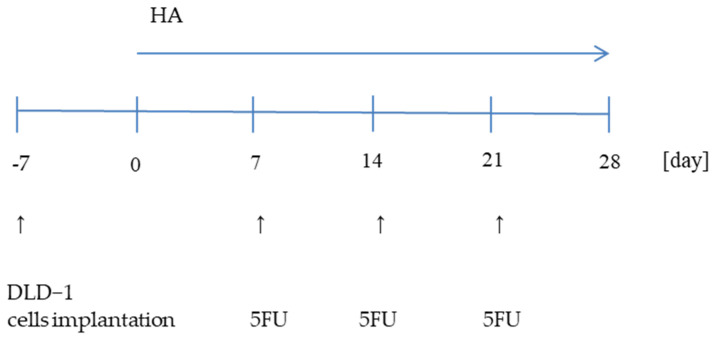
Schematic diagram of the experiment.

Four-week-old NUDE male mice (Cby.Cg.Foxn1<nu>/cmdb) were housed under controlled conditions (21 ± 2 °C, 12 h light/12 h dark cycle) with unlimited access to water. DLD-1 cells (2 × 10^7^) were implanted subcutaneously into the flanks of nude mice. After 7 days, the mice were randomly divided into eight groups (*n* = 9) which were given the HA extract intragastrically once a day for 28 days and/or an intraperitoneal injection of 5FU at a dose of 30 mg/kg b.w. (Sigma-Aldrich, St. Louis, MO, USA) on the 7th, 14th, and 21st day of the experiment (Figure 6).

Eight experimental groups represented:

DLD-1DLD-1 + 30 mg/kg bw 5-FU (DLD-1 + 5-FU)DLD-1 + 1 mg/kg bw of HA extract (DLD-1 + HA1)DLD-1 + 2 mg/kg bw of HA extract (DLD-1 + HA2)DLD-1 + 5 mg/kg bw of HA extract
(DLD-1 + HA5)DLD-1 + 1 mg/kg bw of HA extract +
30 mg/kg bw 5-FU (DLD-1 + HA1 + 5-FU)DLD-1 + 2 mg/kg bw of HA extract +
30 mg/kg bw 5-FU (DLD-1 + HA2 + 5-FU)DLD-1 + 5 mg/kg bw of HA extract +
30 mg/kg bw 5-FU (DLD-1 + HA5 + 5-FU)

The tumor volume was measured every three days using a digital caliper and the orthogonal dimensions (length, width) were determined using the following formula:

$$\mathrm{tumor}\;\mathrm{volume}\;=\;0.5\;\times \;\left(\mathrm{length}\;\times \;{\mathrm{width}}^{2}\right)$$

After 35 days, animals were anesthetized using 4% isoflurane and euthanized. Xenografts were removed and weighed, and blood samples were taken for further examination. Tissues frozen in liquid nitrogen were stored at −80 °C until use.

### 3.4. Immunohistochemistry and H + E Staining

Tissues were fixed in 10% buffered formalin and embedded in paraffin blocks at 56 °C. A histopathological examination was performed using standard hematoxylin-eosin staining. The percentage of the necrotic area was calculated using an Olympus EP50 camera at 40× magnification. Sections of 4 µm thickness were cut and placed onto the silanized glass. The sections were then deparaffinized in xylenes and hydrated in alcohols. The sections were placed in a citrate buffer (pH = 6.0) and incubated in a water bath for 20 min at 98.5 °C to reveal the antigen and then incubated for 20 min at room temperature. The activity of endogenic peroxidase was then blocked by incubation with 3% hydrogen peroxide. Non-specific antibody binding was blocked by horse serum (anti-mouse/rabbit serum produced in Horse, Vector Laboratories, Eching, Germany) for 20 min. The slides were then incubated with a specific antibody—Ki-67 (Biorbyt, Cambridge, United Kingdom, dilution 1:100), for 30 min at room temperature. For the visualization of antibody-antigen binding, a polymer detection system was used (ImmPRESS, Vector Laboratories, Eching, Germany). The reaction products were visualized with diaminobenzidine (DAB, Vector Laboratories, Eching, Germany). The evaluation of immunohistochemical staining was performed with a light microscope using 100× magnification. For immunohistochemical experiments, we used 6 mice per group. Digital images were acquired from 10 different fields, and in each field of view, 100 cancer cells were assessed.

### 3.5. Immunoenzymatic Assays

Apoptotic proteins such as caspase 8, caspase 3, p53, Bcl-2, and survivin were analyzed in mice serum using a commercial ELISA kit. The assays were performed according to the instructions provided by the manufacturer (Cloud-Clone Corp. Unit, Katy, TX, USA). The absorbance was analyzed at 450 nm.

### 3.6. Statistical Analysis

A statistical analysis was performed using Statistica version 13.3 (StatSoft, Tulsa, OK, USA). Intergroup statistical comparisons were analyzed using standard statistical analyses, including one-way analysis of variance (ANOVA) followed by post hoc Duncan’s or Tukey’s comparison test. Differences were considered significant when *p* < 0.05.

## 4. Conclusions

Studies carried out in this work and also by other researchers are only a part of the search for natural substances that could increase the anticancer potential of chemotherapeutics available on the market. The demonstrated effects of HA extracts given alone or with 5FU in the range of tumor growth implies the possibility of its application to optimize the antitumor effect, especially in the range of reducing the adverse effects of 5FU. Moreover, the results showed a significant influence of HA extract on the induction of both pathways of apoptosis in the serum of mice with colon cancer. The obtained beneficial interaction of HA extract with 5FU concerning caspase 3, 8 and p53 protein concentrations seems to be important in diminishing the size of a tumor. It is noteworthy that the effect of the HA extract equivalent to 5FU might be favorable, indicating a potential drug-like effect. All of the above results suggest a need to perform additional detailed studies with the most active fraction to precisely verify the potential synergistic effect of these two compounds.

## Data Availability

Not applicable.

## Supplementary Materials

The following supporting information can be downloaded at: https://www.mdpi.com/article/10.3390/molecules28031352/s1, Table S1. Tumor growth analysis, Figure S1. Violin graph presenting the distribution of tumor volumes of mice in each group. Figure S2. FT-IR spectra of *Heterobasidion annosum* extract. Table S2. Content of selected organic compounds and bioelements in *Heterebasidion annosum* fruiting bodies based on the results from GC-MS, HPLC and F-AAS analysis. TIC–total ion current; d.w.–dry weight. Figure S3. GC-MS chromatogram of chemical composition of *Heterobasidion annosum* fruiting bodies methanolic extract (% TIC, Total Ion Current). Figure S4. HPLC chromatogram of the analyzed indole compounds in *Heterobasidion annosum* extract. Figure S5. HPLC chromatogram of the analyzed sterols in *Heterobasidion annosum* extract.
