# Supplementary material for: Beneficial Proapoptotic Effect of Heterobasidion Annosum Extract in Colorectal Cancer Xenograft Mouse Model
Source: Molecules. 2023 Jan 31;28(3):1352. doi: 10.3390/molecules28031352 (PMC9919637; doi:10.3390/molecules28031352)
Supplement: Supplementary file 1 [file molecules-28-01352-s001.zip › molecules-2157585-supplementary.pdf]

Table S1. Tumor growth analysis. The results are presented as mean ±SD. Table shows statistical significance that were not presented in the main part of manuscript and figures. n(s) – number of mice at the beginning of experiment, n(e) – number of mice at the end of experiment. # p<0.05 DLD-1+HA1+5FU vs DLD-1+HA2+5FU, ### p<0.001 DLD-1+HA1+5FU vs DLD-1+HA2+5FU, ® p<0.05 DLD-1+HA1+5FU vs DLD-1+HA5+5FU, ®®® p<0.001 DLD-1+HA1+5FU vs DLD-1+HA5+5FU.

Day	Tumor size (mm³) - mean ± SD							
	Group							
	**DLD-1**	**DLD-1+5FU**	**DLD-1+HA1**	**DLD-1+HA2**	**DLD-1+HA5**	**DLD-1+HA1+5FU**	**DLD-1+HA2+5FU**	**DLD-1+HA5+5FU**
0	95.09 (±42.24)	63.26 (±30.15)	89.92 (±34.47)	125.96 (±62.17)	120.32 (±30.70)	158.15 (±31.72)	122.60 (±10.61)	60.03 (±35.19)
4	122.46 (±39.32)	58.19 (±36.36)	87.68 (±42.74)	136.08 (±75.34)	146.89 (±66.61)	106.51 (±31.72)	58.71### (±8.03)	54.53®®® (±22.64)
7	169.81 (±90.61)	75.60 (±63.58)	138.47 (±84.99)	197.62 (±144.00)	235.37 (±137.60)	150.57 (±69.15)	74.54 # (±43.87)	87.61 ® (±86.10)
11	294.96 (±156.00)	82.15 (±60.20)	189.03 (±147.63)	225.38 (±181.89)	242.49 (±139.26)	176.55 (±69.54)	84.89 # (±57.58)	114.92 (±144.80)
14	431.50 (±258.48)	143.43 (±89.18)	266.58 (±175.38)	237.73 (±230.88)	307.75 (±177.75)	265.19 (±98.38)	158.56 # (±62.28)	166.92 (±171.43)
18	699.93 (±335.96)	195.68 (±175.92)	319.27 (±269.08)	241.06 (±240.15)	379.24 (±242.43)	299.77 (±130.90)	251.98 (±120.58)	222.72 (±235.73)
21	750.28 (±317.89)	251.00 (±238.45)	376.86 (±328.63)	283.80 (±232.97)	384.91 (±240.01)	368.92 (±160.81)	306.22 (±140.11)	229.78 (±151.67)
24	899.90 (±372.70)	251.18 (±144.36)	452.99 (±399.22)	448.08 (±378.23)	628.67 (±309.95)	578.87 (±149.81)	510.60 (±220.03)	345.81® (±286.51)
28	1098.75 (±482.51)	300.45 (±202.40)	494.77 (±436.59)	510.25 (±513.60)	749.22 (±388.53)	664.91 (±212.91)	545.13 (±333.90)	458.78 (±283.89)
n(s)	9	9	9	9	9	9	9	9
n(e)	7	8	7	7	8	9	9	9

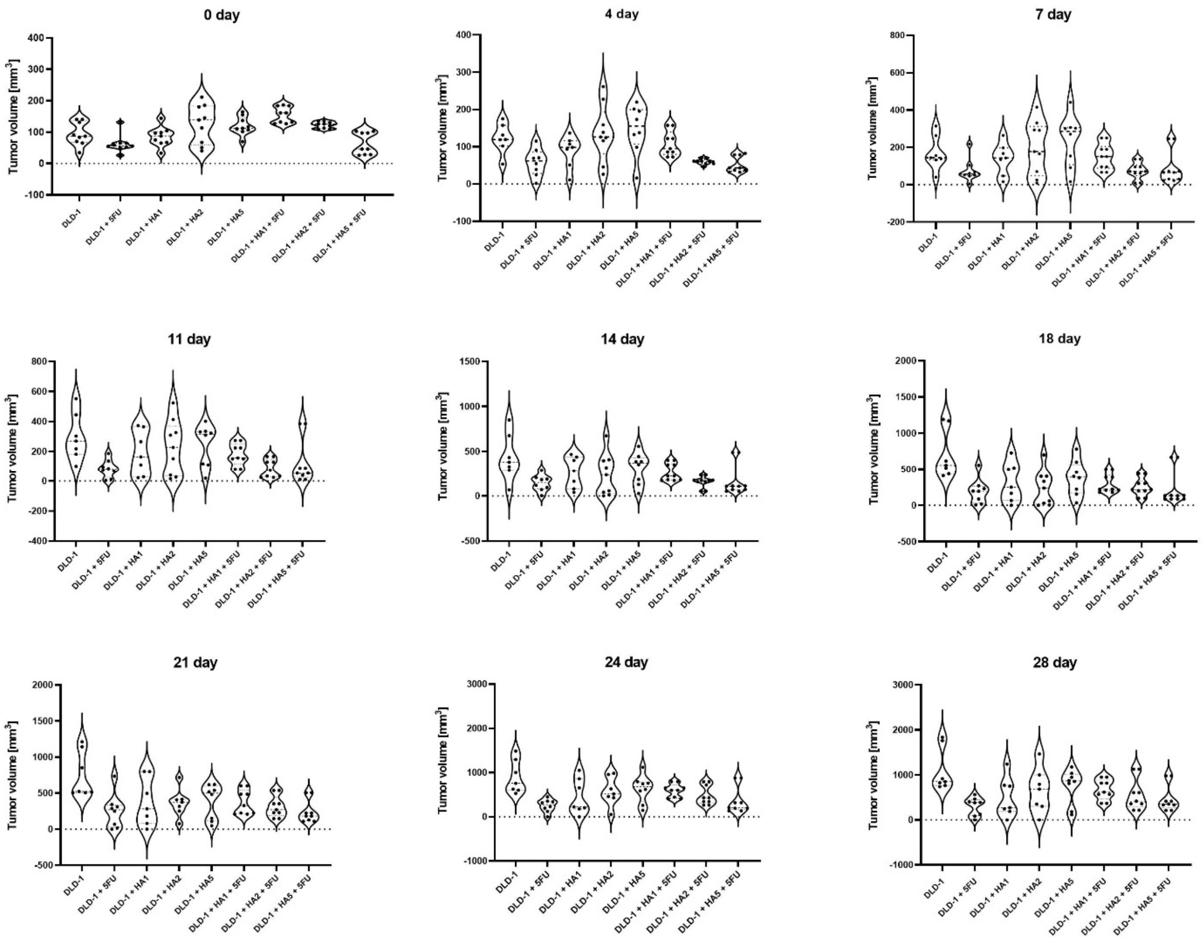

Figure S1. Violin graph presenting the distribution of tumor volumes of mice in each group.

Supplementary data providing chemical composition of the *Heterobasidion annosum* extract (presented in the paper: Sadowska, A.; Zapora, E.; Sawicka, D.; Niemirowicz-Laskowska, K.; Surażyński, A.; Sułkowska-Ziaja, K.; Kała, K.; Stocki, M.; Wołkowycki, M.; Bakier, S.; Pawlik, A.; Jaszek, M.; Muszyńska, B.; Car, H. *Heterobasidion annosum* Induces Apoptosis in DLD-1 Cells and Decreases Colon Cancer Growth in In Vivo Model. *Int. J. Mol. Sci.* 2020, *21*, 3447. https://doi.org/10.3390/ijms21103447)

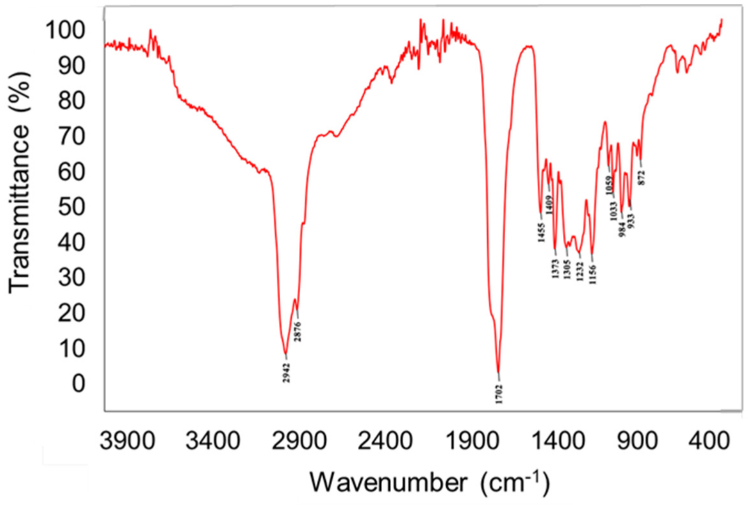

Figure S2. FT-IR spectra of *Heterobasidion annosum* extract.

Table S2. Content of selected organic compounds and bioelements in *Heterebasidion annosum* fruiting bodies based on the results from GC-MS, HPLC and F-AAS analysis. TIC–total ion current; d.w.–dry weight

Groups of compounds	% of TIC
Total carbohydrates	6.82
Total sterols	1.82
Total carboxylic acid	1.74
**Indole compounds**	**[mg/100 g d.w.]±SD**
5-Hydroxy-L-tryptophan	39.1±1.4
L-Tryptophan	34.9±2.4
6-Methyl-D,L-tryptophan	1.1±0.2
Melatonin	*
**Phenolic acids**	**[mg/100 g d.w.]±SD**
Protocatechuic acid	2.2±0,05
Gentisic acid	76.5±0.7
**Sterol compounds**	**[mg/100 g d.w.]± SD**
Ergosterol	9.5±0.07
Ergosterol peroxide	23.8±0.3
**Bioelements**	**[mg/100 g d.w.]±SD**
Cu	1.0±0.06
Fe	14.2±1.7
Zn	4.2±0.3
Mg	186.6±4.5

*less than 0.001 mg/100 g dry weight of extract; n=3.

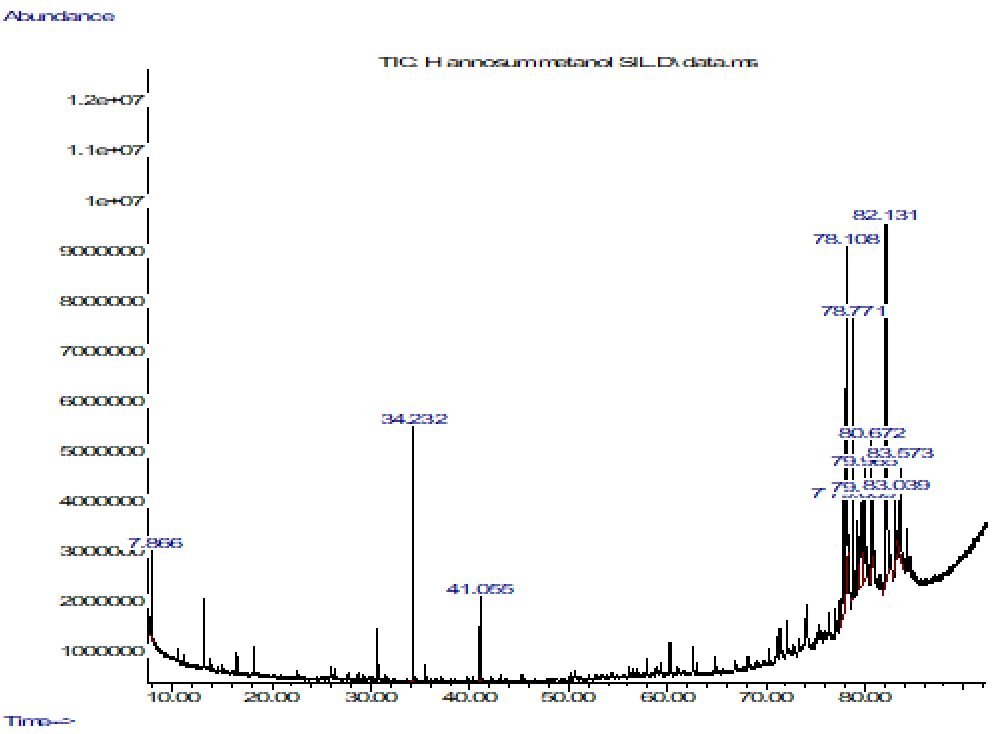

Figure S3. GC-MS chromatogram of chemical composition of *Heterobasidion annosum* fruiting bodies methanolic extract (% TIC, Total Ion Current).

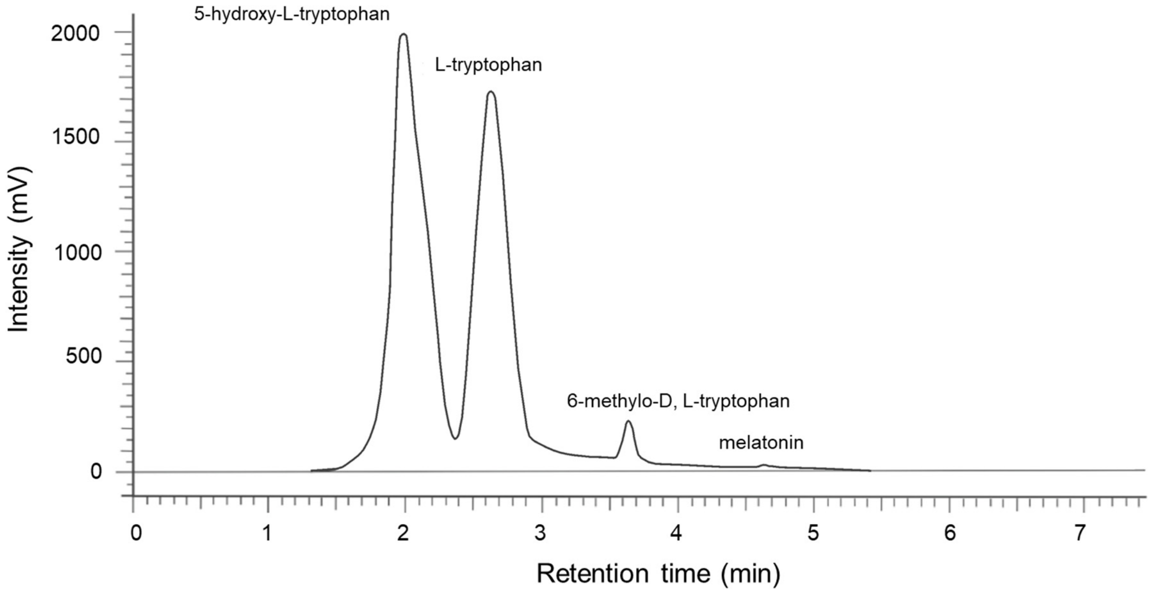

Figure S4. HPLC chromatogram of the analyzed indole compounds in *Heterobasidion annosum* extract.

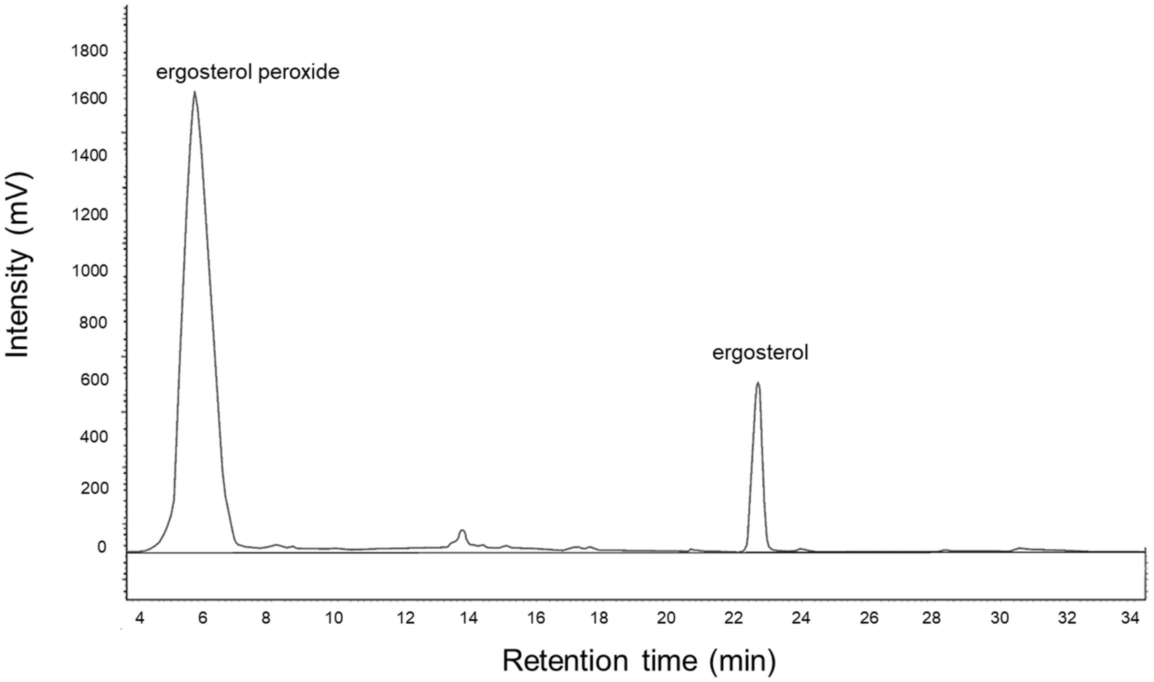

Figure S5. HPLC chromatogram of the analyzed sterols in *Heterobasidion annosum* extract.
